# A Locked Posterior Shoulder Dislocation: An Injury Not to Miss

**DOI:** 10.7759/cureus.66504

**Published:** 2024-08-09

**Authors:** El Mehdi Lahrach, Hamza Skalli, Hamza Benameur, Najib Al Idrissi, Abdeloihab Jaafar

**Affiliations:** 1 Orthopaedics and Traumatology, Cheikh Khalifa International University Hospital, Mohammed VI University of Health Sciences (UM6SS), Casablanca, MAR; 2 Orthopaedics and Traumatology, Avicenna Military Hospital, Marrakesh, MAR; 3 School of Medicine, Laboratory of Genomics, Epigenetics, Personalized and Predictive Medicine, Casablanca, MAR

**Keywords:** mclaughlin technique, surgical intervention, shoulder stability, reverse hill-sachs lesion, locked posterior shoulder dislocations

## Abstract

Locked posterior shoulder dislocations are dislocations that remain unreduced for more than three weeks. In most cases, they are associated with other injuries. We report the case of a 38-year-old male who presented with pain and total functional impotence due to a complex injury, including posterior glenohumeral dislocation, a reverse Hill-Sachs lesion, and a clavicle fracture. Because of the unsuccessful attempts at closed reduction, the patient underwent surgery. We performed the McLaughlin technique, which included the transfer of the subscapularis tendon to the reverse Hill-Sachs lesion, stabilized by bone anchors. At the last follow-up, the patient was doing well and had regained full range of motion with no recurrent dislocation. Clinicians should maintain clinical and radiological suspicion about this injury to timely manage this rare and dangerous injury.

## Introduction

Locked posterior shoulder dislocations are highly uncommon and pose significant challenges in terms of classification, diagnosis, and treatment. The terminology used to describe them can differ in medical literature. These types of dislocations are traditionally characterized as remaining unreduced for at least three weeks [1]. The most common fracture associated with this type of dislocation is the impaction fracture of the humeral head’s articular surface, known as the reverse Hill-Sachs lesion, accounting for 29% of cases, followed by surgical fracture of the neck at 18%. The likelihood of developing reverse Hill-Sachs lesions increases with the patient’s age [1,2].

We outline the typical situation of a patient and an initial clinical situation that led to an inability to reach a diagnosis. In this case, the patient experienced a posterior glenohumeral dislocation, a reverse Hill-Sachs lesion, and a fracture of the outer section of the clavicle, with no associated nerve or circulatory complications.

## Case presentation

**Figure 1:**
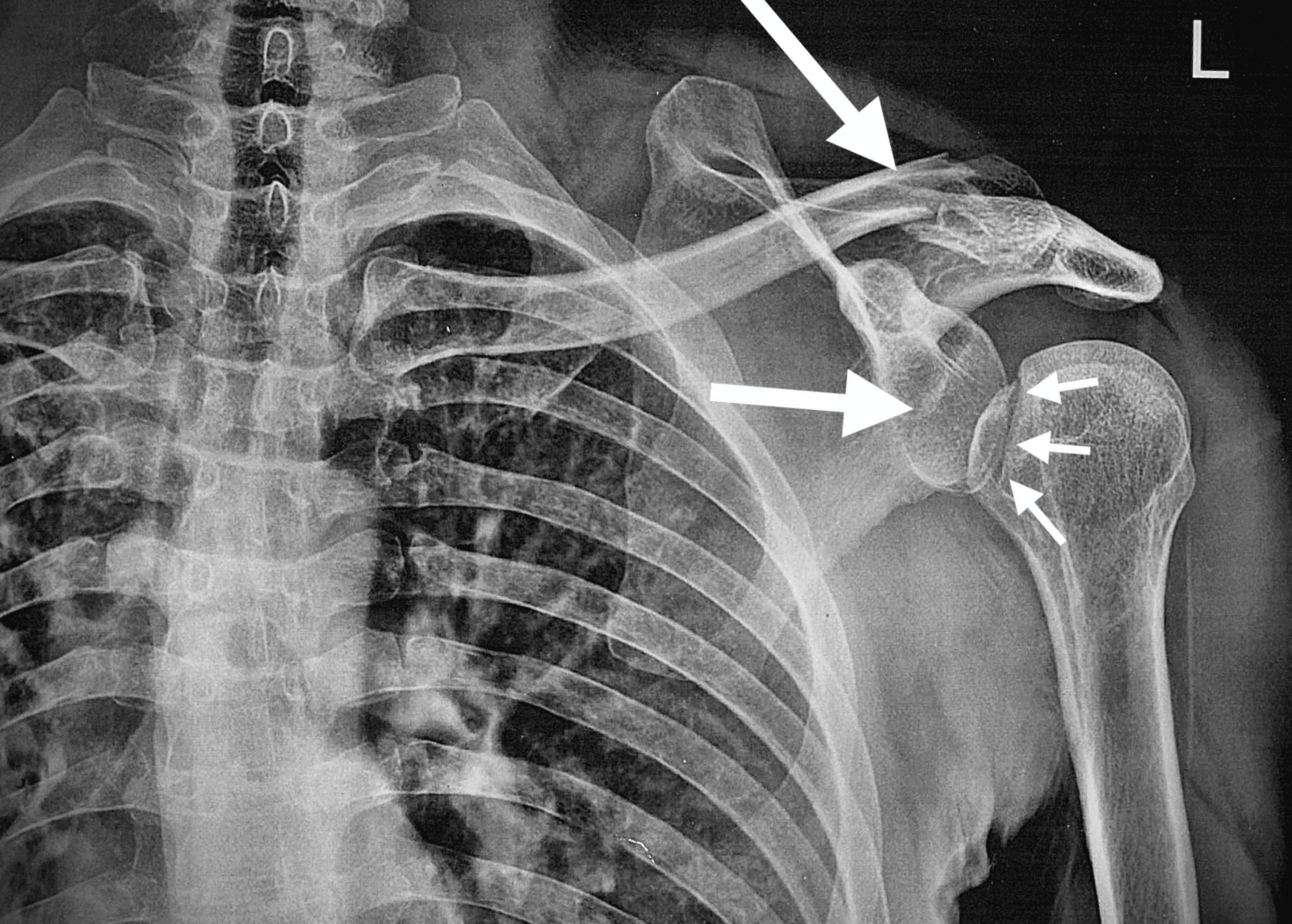
Anteroposterior X-ray of the left shoulder showing the posterior dislocation, fracture in the outer part of the clavicle, and a reverse Hill-Sachs lesion.

**Figure 2:**
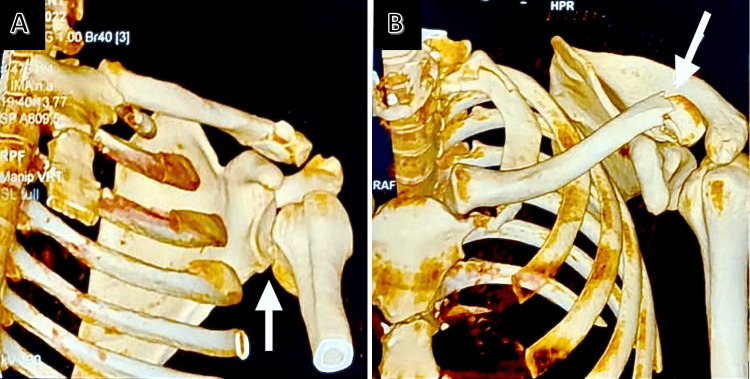
CT scan of the left shoulder showing (A) the joint dislocated toward the back, and (B) a fracture in the outer part of the clavicle.

A 38-year-old patient, with no prior medical history, presented to the emergency department after three weeks for pain and total functional impotence of the left shoulder following a fall. The patient underwent an X-ray of the left shoulder and was discharged with a splint of the shoulder elbow to the body along with oral analgesics. However, the pain worsened at home with the slightest movement, preventing sleep, and leading to a consultation with the orthopedic department. Upon admission, a clinical examination revealed a deformed left upper limb in internal rotation and adduction along with a hematoma on the outer side of the left clavicle. Initial radiological assessment did not reveal any dislocation, but a follow-up appointment a week later confirmed a posterior glenohumeral dislocation, a reverse Hill-Sachs lesion, and a displaced fracture of the outer third of the same clavicle (Figures 1, 2).

**Figure 3:**
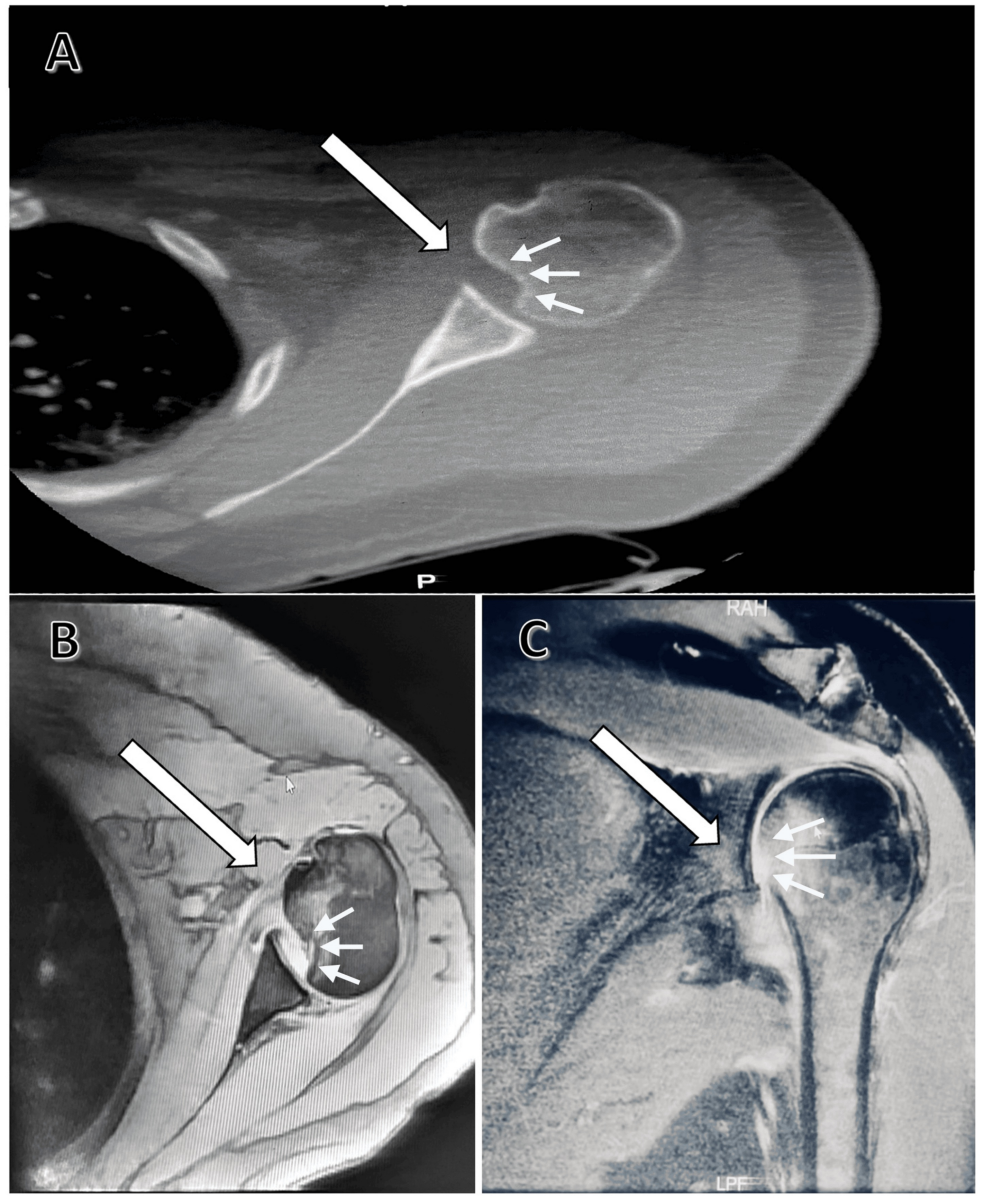
(A) CT scan of the left shoulder showing a reverse Hill-Sachs lesion affecting 25% of the humeral head. (B, C) MRI of the left shoulder showing the extent of the reverse Hill-Sachs lesion.

The dislocation was initially attempted to be reduced under general anesthesia with the elbow immobilized to the body; however, a week later, the radiological results showed that the dislocation had not been resolved. A second attempt to reduce the dislocation under general anesthesia with the shoulder immobilized in neutral rotation was made, but again the dislocation recurred on radiological examination, resulting in a 25% reverse Hill-Sachs lesion. Therefore, surgical treatment was deemed necessary (Figure 3).

**Figure 4:**
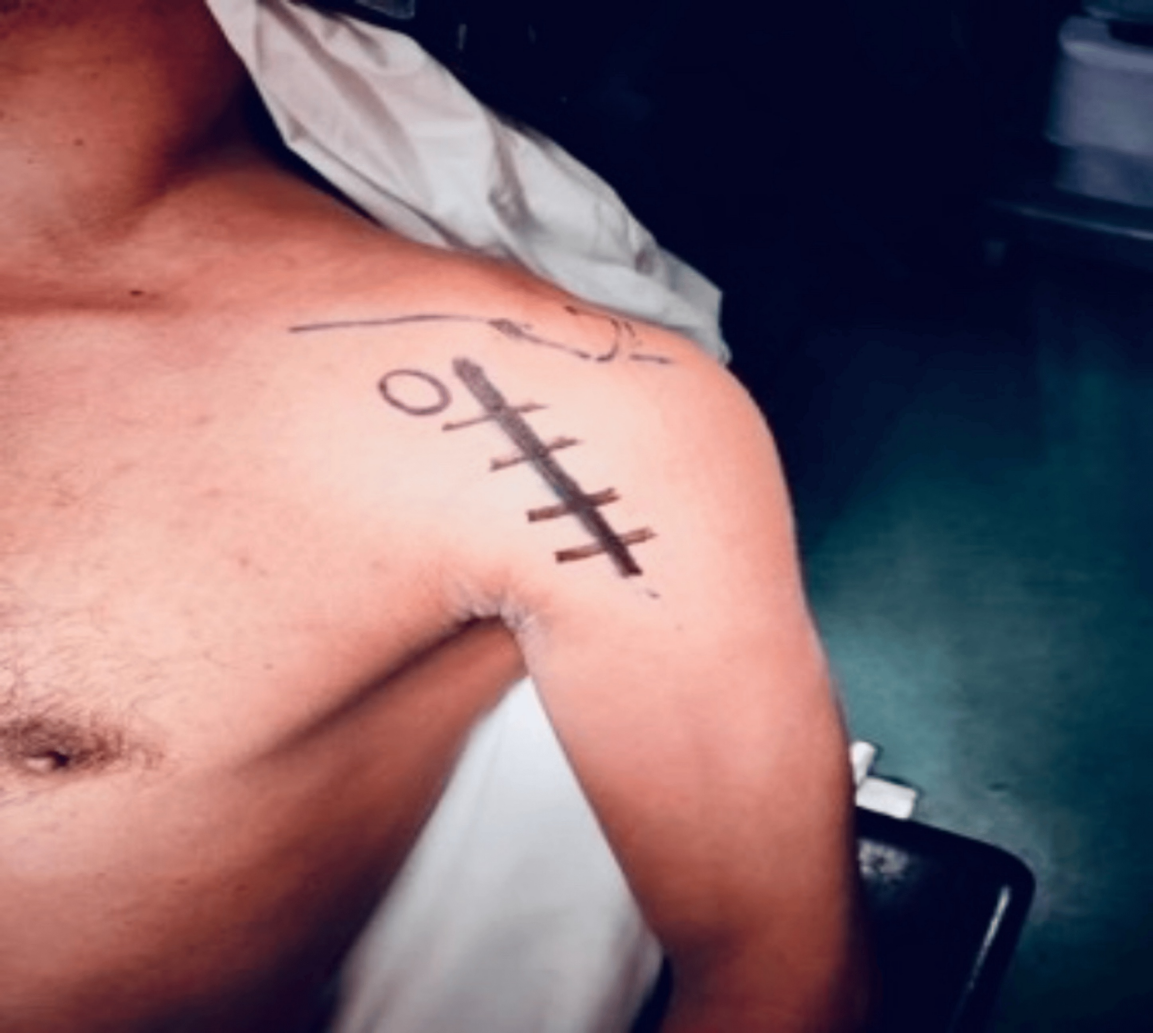
Beach chair position showing the deltopectoral approach.

Under general anesthesia, with the patient in the beach chair position (Figure 4), we performed the McLaughlin method involving repositioning the subscapularis tendon to the left shoulder and stabilizing by bone anchors.

**Figure 5:**
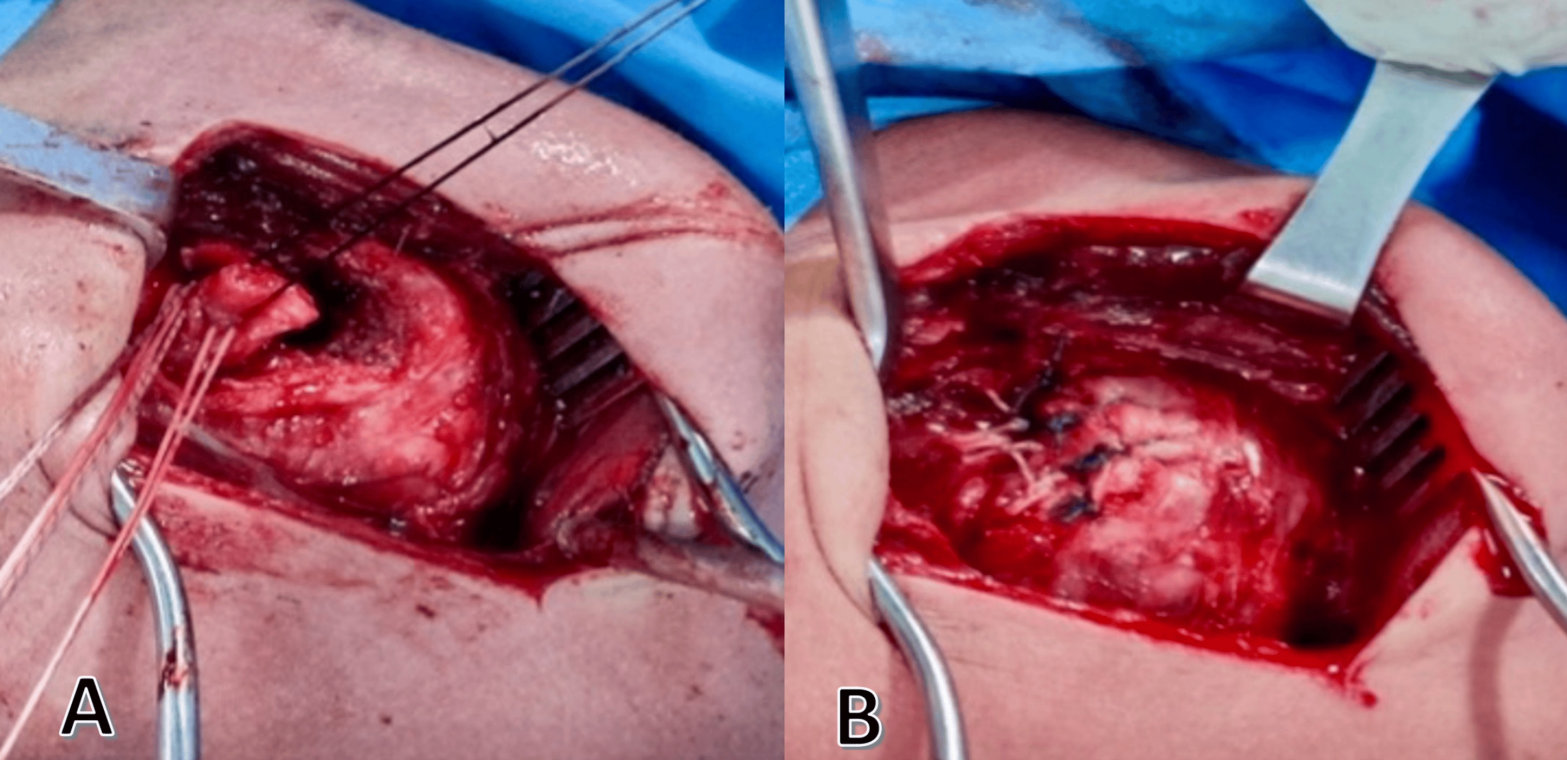
(A) The detachment of the subscapularis tendon from the lesser tuberosity and (B) its subsequent reinsertion utilizing bone anchors.

The reduction was guided with an image intensifier after an incision and deltopectoral dissection. The joint was then opened through a distal incision of the subscapularis tendon. Upon assessing the joint, it was determined that the left shoulder had a 25% deficit, with the long head of the biceps and the glenoid cartilage being normal. Joint irrigation was done, followed by the relocation and reattachment of the subscapularis tendon to the left shoulder using metal anchors (Figure 5).

The conclusion of the surgical procedure involved suturing the end of the subscapularis tendon and closing the rotator cuff. Positive results were obtained from intraoperative stability testing. The surgical site was closed in multiple layers, and the patient’s shoulder was immobilized with an abduction pillow in a neutral rotation, alternating with a specific external rotation splint (Gunslinger’s shoulder) for four weeks, followed by reduced abduction for two weeks. Passive and then active-passive functional rehabilitation started at six weeks.

**Figure 6:**
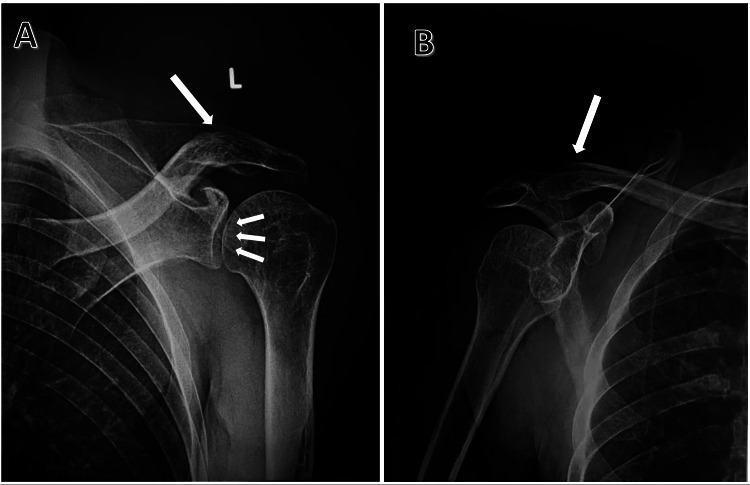
(A) Anteroposterior and (B) lateral X-rays of the left shoulder showing the stable alignment of the glenohumeral joint and the consolidation of the outer portion of the clavicle fracture.

The postoperative X-ray (Figure 6) confirmed the secure positioning of the glenohumeral joint and indicated successful patient recovery. Subsequent treatment proceeded without issue. At the six-month check-up, the patient showed clinical stability and displayed passive shoulder movement. External and internal rotation were measured at 15% and 20%, respectively. Both retroversion and anteversion were at 100% with no persistent discomfort.

## Discussion

Uncovering later-stage glenohumeral dislocations is rare. Terminology varies, with “inveterate dislocations” in French and “locked dislocation” in English. Inveterate dislocation refers to dislocations remaining unreduced after the third week. Successful orthopedic reduction decreases after this time.

Locked posterior dislocations may or may not involve fractures of the proximal humerus or clavicle. These fractures require specific diagnosis and surgical treatment based on age, blood supply, and displacement of the clavicular fracture. The causative elements are comparable to those of recent disruptions, with an increased proportion of seizure episodes or mishaps on public thoroughfares, especially those related to sports [3].

During patient interviews, we often encounter shoulder injuries involving flexion, adduction, and internal rotation with extended elbow. This can cause bone lesions, such as anteromedial humeral head indentation and McLaughlin’s lesion. Lesion size determines treatment options. Other possible issues include glenoid rim indentation/fracture, periosteal appositions, ossification behind the scapular neck, and post-traumatic omarthrosis. Soft tissue injuries may include capsular lesions, biceps incarceration, and supraspinatus tendon rupture during dislocation [4].

From a medical standpoint, a thin individual may display a noticeable protrusion of the coracoid tip beneath the skin, as well as a flattened frontal deltoid. These indications are frequently obscured by swelling. A patient diagnosed with a shoulder contusion keeps the upper limb near the body with the elbow inward and may use a sling for assistance. The scapulothoracic joint permits significant and well-maintained overall flexion, ranging from 80° to 100°, with a range of motion from 70° to 160°. However, there is a reduction in passive or active external rotation, suggesting further assessment for multiple injuries. As a result, the patient is unable to fully rotate the hand upward with the shoulder slightly flexed forward and in neutral rotation, as the shoulder is constrained between 10° and 60° of internal rotation [5].

Radiography confirms the diagnosis and excludes fractures. Scopic centering is recommended for joint space alignment. X-ray findings can be subtle and easily dismissed. The anteroposterior view shows a vertical line on the humeral head, indicating anteromedial indentation. Additionally, there may be a widened glenohumeral joint with an uneven appearance and an empty glenoid cavity. The axillary view is reliable for humeral fractures. Lying down with the arm raised reveals dislocation, notch, and glenoid rim fracture. The Lamy profile confirms misalignment and dislocation. CT plays a critical role in determining the extent of anteromedial indentation on the axillary profile and evaluating the remaining glenoid bone stock. Additionally, MRI may be required for older patients to assess preexisting rotator cuff issues, as posterior dislocations are less likely to cause rotator cuff tears compared to anterior dislocations [6].

Various treatment options for posterior shoulder dislocations exist. Therapeutic abstention is suitable for patients with high functional tolerance. Orthopedic reduction restores the shoulder joint using anesthesia. Immobilization in neutral rotation for three weeks is recommended for stable shoulders. Unstable shoulders should be immobilized in external rotation at 20° for three to six weeks. Open reduction requires surgical stabilization. Posterior stabilization with iliac bone graft is a method similar to Latarjet that requires careful attention to avoid hardware protrusion. An alternative approach is posterior arthroscopic stabilization, which was suggested by a singular case study with a diagnostic delay of four weeks and entails an arthroscopic procedure [7].

The procedure known as McLaughlin subscapularis transfer involves the detachment of the subscapularis from the lesser tuberosity and its reinsertion into the anteromedial indentation using either transosseous sutures or bone anchors [8].

The shoulder prosthesis can be limited by instability. In cases where external rotation for head osteotomy is not possible, an anatomical total shoulder prosthesis is recommended. This involves a second posterior approach, cutting the infraspinatus and posterior capsule to visualize the head, performing the osteotomy, and extracting the head. Factors such as age, functional needs, dislocation length, and humeral head indentation determine the approach, emphasizing the importance of preoperative CT scans. Categorizing indentation and the presence of osteoarthritis helps determine the appropriate treatment [9].

Therapeutic abstention could be recommended for elderly patients with medical conditions, individuals with unstable epilepsy, or instances with minor shoulder range of motion loss [10].

It is advisable to perform orthopedic reduction for indentations that are smaller than 25% in circumference. Surgical stabilization is the recommended procedure for indentations ranging from 25% to 45% to prevent recurrence. In cases where the indentation is greater than 45% and there is no glenoid lesion, a humeral prosthesis is the appropriate course of action. If the indentation exceeds 45% and there are lesions present, it is recommended to use a complete shoulder prosthesis without rotator cuff tear or retroversion. For cases involving indentations, lesions, and tears in older patients, a reverse complete shoulder prosthesis may be advantageous [11].

The frequent concerns revolve around the repetitive occurrence of posterior dislocation, osteonecrosis, and post-traumatic osteoarthritis.

## Conclusions

Locked posterior shoulder dislocations require an accurate diagnosis for effective treatment. Early identification and intervention are critical to prevent complications. Misdiagnosis leads to prolonged discomfort and the need for invasive surgery. The McLaughlin method demonstrated the effectiveness of surgical intervention when conservative measures fail. Successful reduction and stabilization of the shoulder, along with rehabilitation, resulted in satisfactory recovery.
